# Prime Editing Modification with FEN1 Improves F508del Variant Editing in the *CFTR* Gene in Airway Basal Cells

**DOI:** 10.3390/ijms26167943

**Published:** 2025-08-18

**Authors:** Olga V. Volodina, Anna G. Demchenko, Arina A. Anuchina, Oxana P. Ryzhkova, Valeriia A. Kovalskaya, Ekaterina V. Kondrateva, Ekaterina V. Artemova, Vyacheslav Y. Tabakov, Maxim A. Ignatov, Natalia Y. Vorobyeva, Andreyan N. Osipov, Alexander V. Lavrov, Svetlana A. Smirnikhina

**Affiliations:** 1 Laboratory of Genome Editing, Research Centre for Medical Genetics, 115522 Moscow, Russia; olgavolodina@med-gen.ru (O.V.V.);; 2 Laboratory for Structural Analysis and Engineering of Membrane Systems, The Research Center for Molecular Mechanisms of Aging and Age-Related Diseases, Moscow Institute of Physics and Technology, 141701 Moscow, Russia; 3 The Shared Resource Centre “Genome”, Research Centre for Medical Genetics, 115522 Moscow, Russia; 4 The Shared Resource Centre “Biobank”, Research Centre for Medical Genetics, 115522 Moscow, Russia; 5 Laboratory of Radiation Biophysics, Department of Experimental Radiobiology and Radiation Medicine, State Research Center-Burnasyan Federal Medical Biophysical Center of Federal Medical Biological Agency (SRC-FMBC), 123098 Moscow, Russia

**Keywords:** prime editing, gene editing, gene therapy, cystic fibrosis, FEN1, EXO1

## Abstract

Prime editing is a promising approach for correcting pathogenic variants, but its efficiency remains variable across genomic contexts. Here, we systematically evaluated 12 modifications of the PEmax system for correcting the *CFTR* F508del pathogenic variant that caused cystic fibrosis in patient-derived airway basal cells. We chose EXO1 and FEN1 nucleases to improve the original system. While all tested variants showed comparatively low efficiency in this AT-rich genomic region, 4-FEN modification demonstrated significantly improved editing rates (up to 2.13 fold) compared to standard PEmax. Our results highlight two key findings: first, the persistent challenge of AT-rich target sequence correction even with optimized editors, and second, the performance of 4-FEN suggests its potential value for other genomic targets.

## 1. Introduction

Cystic fibrosis is an autosomal recessive disease caused by several pathogenic variants in the *CFTR* gene [1]. The main cause of cystic fibrosis in European and Russian populations is the F508del pathogenic variant in the *CFTR* gene [2,3]. It leads to disruption of CFTR protein folding, preventing the protein from migrating to the cell membrane. As a result, chloride ions (Cl^−^) cannot pass through the membrane, leading to thick mucus accumulation on cell surfaces [1]. Patients with cystic fibrosis die at a young age due to severe lung infections caused by mucus obstruction and recurrent inflammation [4]. While pathogenic therapy for cystic fibrosis exists, it is lifelong, expensive, and associated with several serious side effects [5,6,7]. Therefore, developing causal therapies targeting the genetic basis of cystic fibrosis, such as correcting the F508del mutation directly, remains essential.

For developing causal therapy for cystic fibrosis, we chose the prime editing platform [8]. It is based on the CRISPR/Cas9 system but does not introduce double-strand DNA breaks, making PE potentially safer than CRISPR/Cas9 due to low off-target activity. It has also shown higher efficiency in several studies [9].

**Figure 1:**
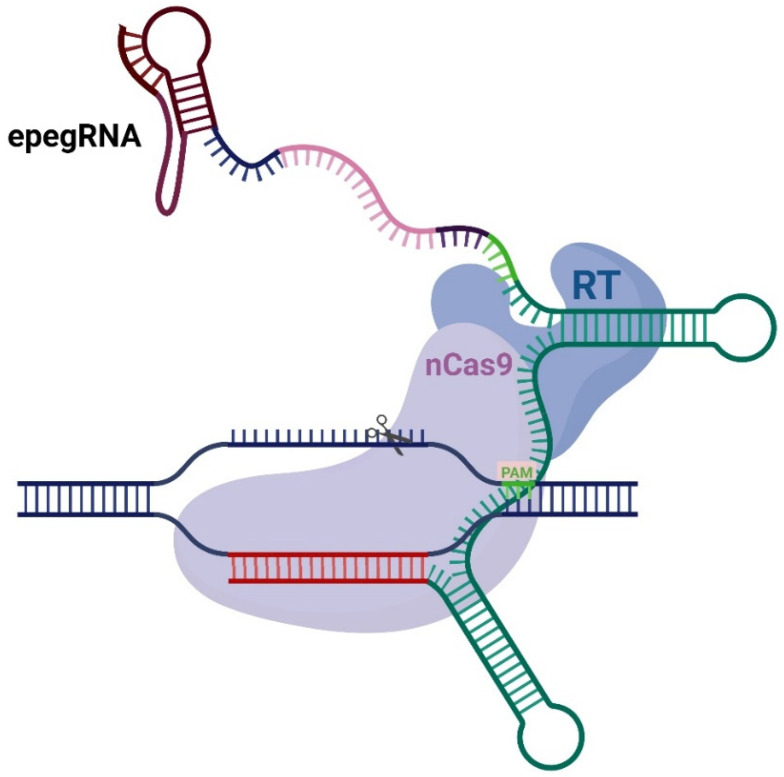
Schematic representation of the prime editing system. Standard prime editor consists of nickase Cas9 (nCas9), reverse transcriptase (RT), and engineered pegRNA (epegRNA). epegRNA consists of tevopreQ_1_ motif (burgundy), linker (dark blue), PBS (pink), RTT (green and dark violet), spacer (red) and scaffold (aqua). PAM is indicated in light green.

Prime editing is a relatively new gene editing platform where the editor consists of nickase Cas9 (instead of nuclease) fused to reverse transcriptase MMLV-RT and pegRNA—a guide RNA for prime editing (Figure 1).

**Figure 2:**
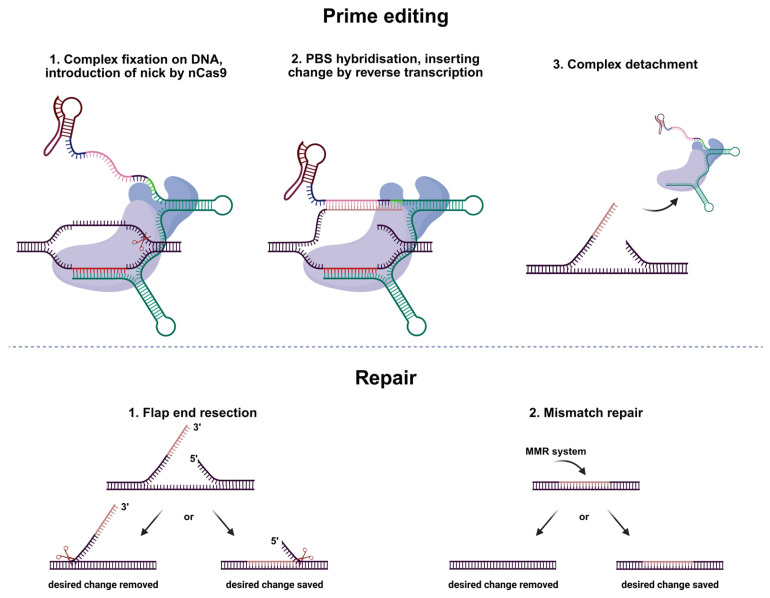
Schematic representation of prime editing and following repair pathways.

Editing consists of several steps. First, the spacer binds to complementary genomic DNA. Then, nCas9 introduces a nick three nucleotides away from the PAM. The flapping single strand hybridizes with the primer-binding site of pegRNA and undergoes reverse transcription using the reverse transcriptase template site of pegRNA. After the editing complex detaches, the change must remain intact during two repair steps. First, flap end resection removes the unedited 5′ flap—if this fails, the edit is lost. Then, the mismatch repair system may still excise the edit. The change only becomes permanent if it escapes both repair processes (Figure 2) [10].

For our study, we chose PEmax, which is the most efficient platform without additional factors such as MLH1dn or sgRNAs [8], which is important for therapy development, as non-targeted sgRNAs lead to indels in the editing locus and MLH1dn can disrupt the DNA repair process. We also used epegRNAs (engineered pegRNAs); they have a structured RNA motif called tevopreQ1, unlike original pegRNAs. This motif protects the 3′-end from degradation, which is crucial for successful editing, as PBS and RTT sites are located there [11].

**Figure 3:**
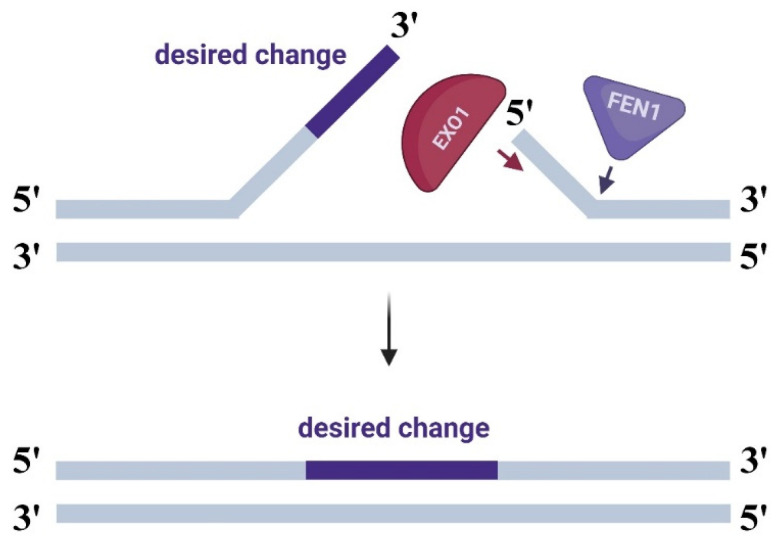
Highlighting the roles of EXO1 and FEN1 nucleases in flap end resection.

An approach we examine in this study involves enhancing the system through modifications to the PEmax editor complex. We decided to fuse PEmax with flap endonuclease (FEN1) and exonuclease 1 (EXO1) (a full-length nuclease and an active domain—HEX-N2) using 4- or 16-amino acid linkers. During flap end resection in the repair phase after prime editing, these nucleases remove the 5′ single strand lacking the desired edit, promoting hybridization of the edited strand with the gDNA (Figure 3) [10].

To enhance prime editing efficiency for the F508del variant in *CFTR*, we designed and tested 12 modified versions of the PEmax system. Through comparative analysis, we identified the 4-FEN modification as the most impactful, demonstrating a 2.13-fold enhancement in editing efficiency relative to the unmodified PEmax system. This result highlights the potential of engineered fusion architectures to improve PE efficacy for therapeutic applications.

## 2. Results

### 2.1. Primary Screening of All the Modifications

**Figure 4:**
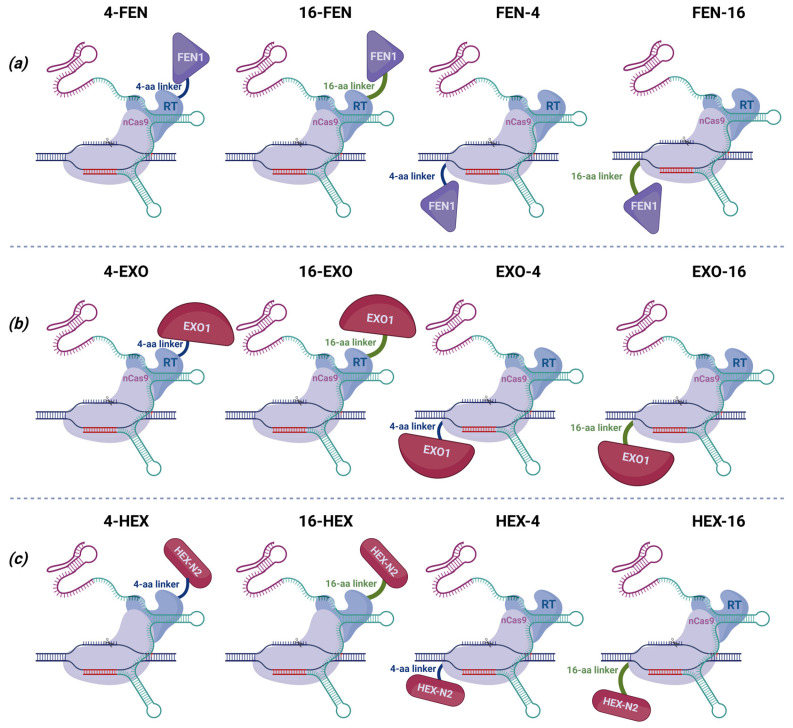
Schematic representation of prime editor modifications: (**a**) all variants with nuclease FEN1 (4-aa or 16-aa linker, fused to either the C-terminus of RT or N-terminus of Cas9), (**b**) all variants with nuclease EXO1 (4-aa or 16-aa linker, fused to either the C-terminus of RT or N-terminus of Cas9), and (**c**) all variants with nuclease HEX-N2 (4-aa or 16-aa linker, fused to either the C-terminus of RT or N-terminus of Cas9).

To determine whether the addition of exonuclease enhances the effectiveness of prime editing, we have developed 12 genetic constructs. Co-transfection of unfused nucleases would be unsafe, as increasing their amount could lead to unpredictable consequences in the genome. For these reasons, we fused both nucleases with PEmax via 4- or 16-amino acid linkers, which have been successfully used in protein fusion in base editors—another CRISPR/Cas9-based system that introduces single-nucleotide substitutions [12,13]. We fused nucleases with the system either at the N-terminus of nCas9 or at the C-terminus of RT, as their optimal conformation for efficiency was hard to predict in silico. We named them accordingly as the nuclease and linker location (Figure 4).

Initial testing of 12 modified systems was conducted in two different patient-derived basal cell lines with homozygous F508del variant in the *CFTR* gene. Eleven modifications were evaluated with epegRNA#1, while the twelfth (16-FEN) was excluded from initial analysis due to technical constraints. This variant was tested with epegRNA#2 in parallel validation experiments, as accumulated data indicated epegRNA#1’s inconsistent performance. Other experimental properties were not changed. All the results were evaluated statistically using Dunn’s test, but only one modification showed significant efficacy, so we relied on mean editing efficiency and Cliff’s delta to determine modifications for further testing.

#### 2.1.1. Modifications with FEN1 Nuclease

**Figure 5:**
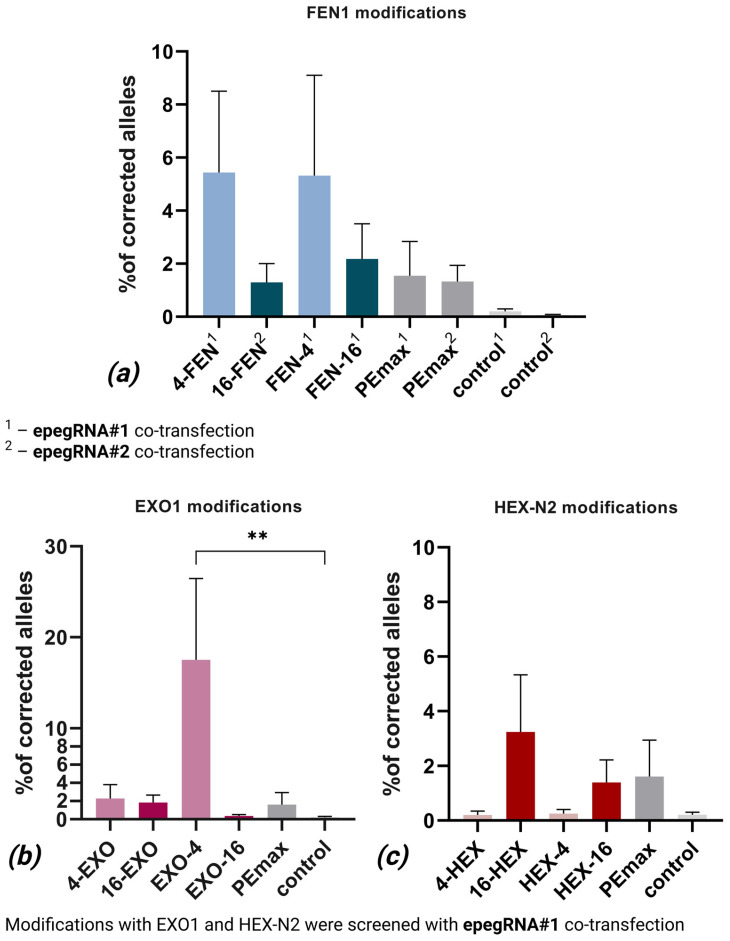
Editing efficiency of modified PEmax variants in patient-derived basal cells: (**a**) FEN1 variants tested with both epegRNA#1 (labeled ‘1’) and epegRNA#2 (labeled ‘2’), showing mean editing efficiency ± SEM (n = 4–6). Includes PEmax positive control (1.55% for epegRNA#1, 1.33% for epegRNA#2) and negative controls; (**b**) EXO1 variants tested with epegRNA#1, displaying mean editing efficiency ± SEM (n = 6). Contains PEmax control (1.55%) and negative control; and (**c**) HEX-N2 variants tested with epegRNA#1, presenting mean editing efficiency ± SEM (n = 6). Includes PEmax control (1.55%) and negative control. All panels represent data from two independent biological replicates (cell lines from patients P1 and P7). **—statistical significance with *p* < 0.01.

Modifications with 4-FEN and FEN-4 demonstrated higher mean editing efficiencies normalized for the transfection efficiency (5.44% and 5.32%, respectively, by deep target sequencing of edited locus) compared to 16-FEN and FEN-16 (1.29% and 2.18%, respectively). While Dunn’s test did not reveal statistically significant differences versus negative control (*p* > 0.05), Cliff’s Delta analysis indicated large effect sizes for 4-FEN (Δ = 0.75) and 16-FEN (Δ = 0.69) and medium effect sizes for FEN-4 (Δ = 0.42) and FEN-16 (Δ = 0.39). These results suggest that shorter linkers may work better for FEN1 modifications (Figure 5a).

#### 2.1.2. Modifications with EXO1 Nuclease

EXO1 modifications did not show a consistent pattern, with only EXO-4 showing a statistically significant effect with editing efficiency normalized for the transfection efficiency of 17.45% (*p* = 0.005). Other EXO1-based variants did not show either high or significant edition rates, though 4-EXO and 16-EXO performed with mean efficiency of 2.27% and 1.8%, respectively, and showed medium and large effects judging by Cliff’s delta (0.42 and 0.52, respectively), while EXO-16’s correction rate was only 0.35, and this modification had a small effect (Δ = 0.22). While being the only group with statistically significant modification, EXO1-based variants showed high variability in results, with no apparent influence of linker length (Figure 5b).

#### 2.1.3. Modifications with the HEX-N2 Domain of EXO1 Nuclease

Modifications 16-HEX and HEX-16 demonstrated higher mean editing efficiencies normalized for the transfection efficiency (3.15% and 1.36%, respectively) compared to 4-HEX and HEX-4 (0.16% and 0.24%). While Dunn’s test did not reveal statistically significant differences versus controls (*p* > 0.05), Cliff’s Delta analysis indicated small effect sizes for 16-HEX and HEX-16 (Δ = 0.25 and Δ = 0.28, respectively) and negligible effects for 4-HEX and HEX-4 modifications. We assume that longer linkers may be preferable for HEX-N2-based modifications (Figure 5c).

For comparison, the PEmax control in these initial experiments (epegRNA#1 with 11 modifications) showed 1.55% editing efficiency with a medium Cliff’s delta effect (Δ = 0.36). In the experiments with the final modification tested with epegRNA#2 PEmax, it demonstrated 1.33% efficiency and a large effect size (Δ = 0.58).

Based on our initial evaluation, we selected three modifications for further characterization. First, EXO-4 is the variant with the highest correction rate (17.45%) and the only modification with statistically significant correction efficiency. Second, 4-FEN, with both a large Cliff’s delta effect (Δ = 0.72) and quite high 5.44% editing efficiency. Lastly, FEN-4 demonstrated promising editing rates (5.32%) and a medium Cliff’s delta Δ = 0.38. While 16-EXO and 16-FEN modifications showed larger effect sizes (Δ = 0.52 and Δ = 0.69), compared to FEN-4, they were excluded due to low absolute editing efficiency (1.8% and 1.29%). The observed variability between technical replicates prompted our transition to epegRNA#2 for all subsequent validation experiments.

### 2.2. In-Depth Analysis of the Most Efficient Modifications

**Figure 6:**
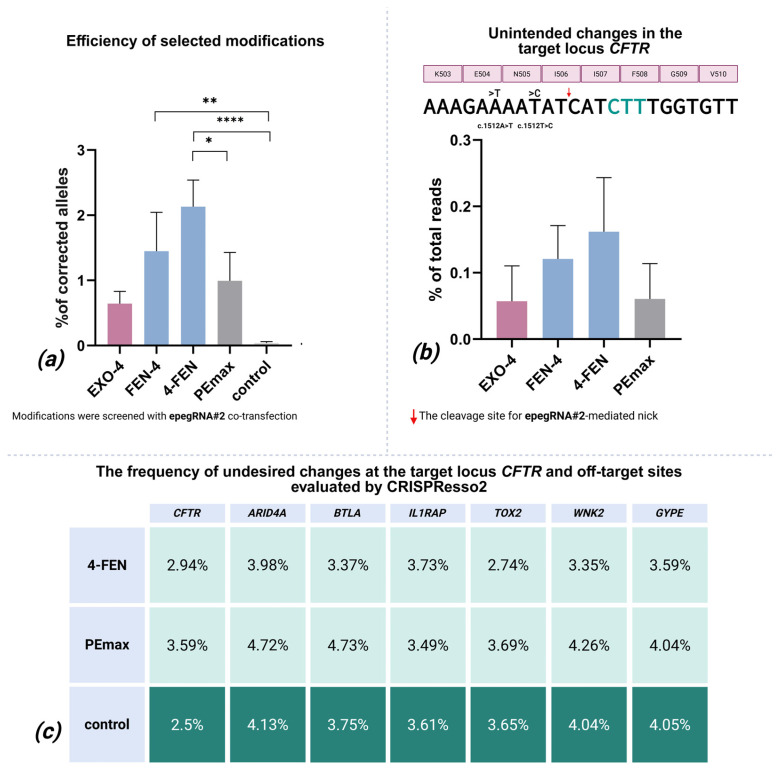
Editing efficiency and precision of selected prime editor modifications: (**a**) percentage of target alleles successfully corrected for each modified editor variant and (**b**) frequency of unintended variants in edited reads across experimental conditions. Pink rectangles represent amino acids of the CFTR protein, green nucleotides represent desired insert. Error bars represent SEM (n = 9); * *p* < 0.05, ** *p* < 0.01, **** *p* < 0.0001. (**c**) The frequency (%) of unintended insertions, deletions (indels), and substitutions at the target and off-target sites, assessed using CRISPResso2 analysis without further analysis and normalization for the transfection efficiency (n = 2).

In-depth analysis of top candidates (4-FEN, FEN-4, and EXO-4) using epegRNA#2 in three patient-derived airway basal cell lines with the homozygous F508del variant in the *CFTR* gene showed different performance patterns. While 4-FEN (2.13%) and FEN-4 (1.45%), both normalized for the transfection efficiency, showed statistically significant differences from negative controls (*p* < 0.0001 and *p* = 0.0093, respectively), the previously successful EXO-4 modification efficiency (0.6%) was not significantly distinct from negative control. PEmax also did not demonstrate high efficiency (1%) or a statistically significant difference with the control. Notably, editing efficiencies were consistently lower than in primary screening, though with reduced variability. Dunn’s test also confirmed 4-FEN’s superiority over PEmax (*p* = 0.03), while Cliff’s Delta revealed large effects for all test modifications (vs. PEmax’s medium effect Δ = 0.41), establishing 4-FEN as the most efficient modification among those tested (Figure 6a).

### 2.3. Indels and Mutagenesis in the Editing Locus

We systematically evaluated undesired editing events at the target locus across all selected modifications. After technical errors were removed from the analysis, no deletions or additional insertions were detected at the target locus. During SNV (single nucleotide variants) evaluation, statistical analysis of overall read sum between edited samples and the negative control group revealed significant differences in mutation frequencies between edited (FEN-4 and PEmax) and control samples (Dunn’s test, *p* < 0.05) and the absence of significant differences in SNV frequencies between EXO-4, 4-FEN, and control samples (Dunn’s test, *p* > 0.05). However, we analyzed mutation content, and all variant alleles detected in edited samples were also present in control datasets, with no editing-specific mutations identified. After applying our filtering criteria (99th percentile of non-transfected control frequencies as threshold), the remaining variants showed no reproducible pattern across biological/technical replicates. These observations, combined with the absence of editing-unique mutations, suggest the detected variants likely represent technical artifacts (e.g., NGS errors, PCR amplification biases, or spontaneous mutagenesis during cell cultivation) rather than genuine undesired effects of the editing systems.

### 2.4. Undesired Changes in Edited Reads

Within successfully edited reads, we identified two consistent editing-associated substitutions (c.1512A>T and c.1515T>C) that consistently appeared simultaneously. Quantitative analysis showed that these unintended mutations appeared at mean normalized for the transfection efficiency frequencies of 0.16% of total reads for 4-FEN, 0.12% for FEN-4, and 0.06% for both EXO-4 and standard PEmax systems (Figure 6b). Those substitutions appeared in the non-transfected controls, but not simultaneously.

This analysis confirms that both conventional and modified prime editors maintain high specificity, with unintended mutations being restricted to predictable locations adjacent to the edit site and occurring at frequencies below 0.5%. The technical noise inherent to deep sequencing protocols accounts for the majority of observed sequence variations.

### 2.5. Off-Target Effects

We performed off-target analysis exclusively on the 4-FEN editing system—our lead candidate—which revealed no detectable unintended DNA changes. As a positive control, we evaluated samples edited with the PEmax and as a negative control, non-transfected samples. Potential off-target sites were selected based on partial spacer homology (allowing two non-consecutive mismatches and single-nucleotide bulges) and proximity to NGG PAM sequences, with prioritization of tumor-associated loci. Targeted deep sequencing of intronic regions in genes *ARID4A, BTLA, IL1RAP, TOX2, WNK2,* and *GYPE* showed no significant increase in indels or single-nucleotide variants in 4-FEN-edited samples compared to PEmax and non-transfected controls (Dunn’s test, *p* > 0.05 for all loci). Figure 6c shows that even raw CRISPResso data—without additional filtering—reveals minimal to no difference between 4-FEN-edited samples and negative controls. After excluding PCR artifacts, variant frequencies in the edited samples remained at baseline control levels; the content of the changes was not different from control either. This data demonstrates that the 4-FEN system maintains high editing specificity without introducing measurable off-target mutations at predicted sensitive sites.

### 2.6. Quantification of γH2AX Foci

**Figure 7:**
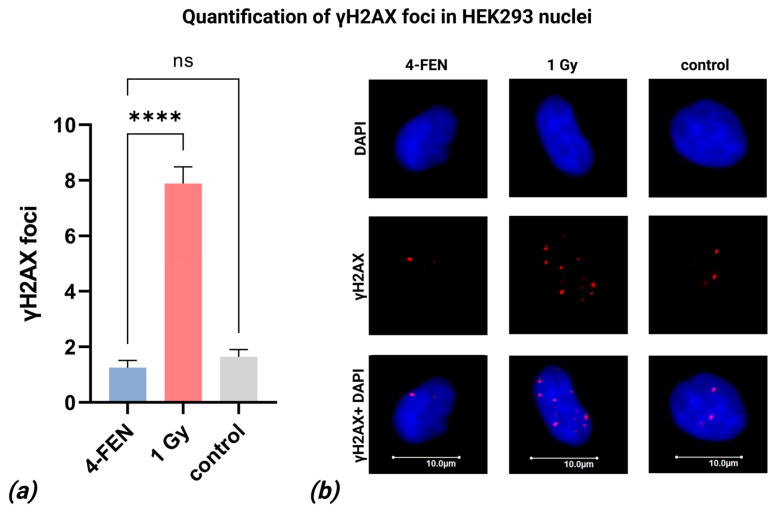
Quantification of γH2AX foci: (**a**) frequency of γH2AX foci in HEK293T cells after lipofection with irradiated cells as a positive control and non-transfected cells as a negative control. Error bars represent SEM (n = 31–36); **** *p* < 0.0001; ns—non significant. (**b**) Microphotographs of immunofluorescently stained transfected, irradiated, and control HEK293T cells showing γH2AX (red) foci. DAPI counterstaining is shown in blue.

Given that FEN1 overexpression has been associated with genomic instability and increased γH2AX foci formation, we assessed the number of γH2AX foci in HEK293T cells following lipofection with the 4-FEN editing system. Transfection efficiency was 99.87% on average. Cells irradiated with 1 Gy served as a positive control, showing an expected elevation in γH2AX foci (7.89 foci/nucleus on average, Figure 7). In contrast, both untransfected control cells and 4-FEN-lipofected cells showed minimal foci numbers (1.64 and 1.26 foci/nucleus on average, respectively; Figure 7). A statistically significant difference in γH2AX foci counts was observed between 4-FEN-lipofected cells and 1 Gy-irradiated positive controls (*p* < 0.0001), whereas no significant difference was detected between lipofected cells and untreated negative controls (*p* > 0.05) (Figure 7a). These results further support the conclusion that the 4-FEN system does not induce detectable DNA double-strand breaks under the tested conditions.

## 3. Discussion

In this study, we systematically evaluated 12 modifications of the prime editing system by fusing DNA repair nucleases to the PEmax system. Specifically, we fused FEN1, EXO1, and a truncated EXO1 variant (HEX-N2 domain) to the PEmax using either 16-amino acid or 4-amino acid linkers as part of our therapeutic development strategy for cystic fibrosis treatment. Each configuration was tested in multiple spatial orientations relative to PEmax, with preliminary screening identifying lead candidates for subsequent in-depth characterization.

FEN1 is a key nuclease involved in DNA replication and repair processes. As a member of the Rad2/XPG family, FEN1 plays critical roles in processing 5′ overhanging flaps during DNA repair and Okazaki fragment maturation during lagging strand DNA synthesis. This nuclease demonstrates great efficiency in processing short 5′ overhangs (<20–30 nt) through its tracking mechanism, while longer flaps require cooperative action with DNA2 helicase. FEN1’s activity is particularly important for maintaining genomic stability at repetitive sequences, where DNA secondary structures can inhibit its protective function, potentially leading to trinucleotide expansions. The enzyme forms functional complexes with various replication and repair proteins, including PCNA and DNA polymerase δ, which can process very short flaps (1–2 nt) even in FEN1’s absence, though with limited capacity [14,15]. EXO1 represents another crucial member of the Rad2/XPG nuclease family with multifaceted roles in DNA metabolism. This enzyme participates in mismatch repair, homologous recombination, and DNA replication through its 5′→3′ exonuclease and flap endonuclease activities. EXO1 interacts with numerous repair proteins, including MLH1, MSH2, and the MRN complex, with its activity being regulated by multiple factors such as RPA and 14-3-3 proteins. The 395-amino acid N-terminal fragment (HEX-N2), containing the catalytically active exonuclease domain, has been particularly well-characterized through crystallographic studies and retains significant biochemical activity. EXO1 demonstrates substrate versatility, processing double-stranded DNA, nicks, gaps, and even resolving Holliday junctions. Its function often overlaps with and complements FEN1 activity, particularly in DNA replication, where it can serve as a backup for Okazaki fragment processing, as evidenced by studies in *S. cerevisiae* [15]. We chose EXO1 and FEN1, as they take part in the repair process of prime editing, helping to save the desired correction.

For this study, we selected 4-amino acid (aa) and 16-aa linkers, which have previously proven effective in constructing diverse Cas9 fusions [12,13].

Even though several versions of prime editing have been developed, we did not choose larger constructs, as they do not provide significantly higher efficiency while being harder to deliver. They are also harder to modify due to their size, and system modification was part of our study plan.

Lipofection was employed throughout our experiments, with transfection efficiency monitored indirectly via transfection of size-matched eGFP plasmid at the adjacent plate cells during the experiment. Given the documented challenges of transfecting basal cells with large plasmids [16], we avoided further size expansion through eGFP cassette insertion to prevent additional efficiency loss. All reported data were normalized to account for transfection variability.

Primary screening showed different patterns for FEN1 and HEX-N2 modifications, where linker length (4-aa for FEN1 vs. 16-aa for HEX-N2) proved to be more important than nuclease positioning relative to PEmax. While Spearman and Kendall’s tau tests showed no statistically significant correlations (*p* > 0.05), Cliff’s delta effect size measures supported these observations. No clear trends were observed for EXO1 configurations regarding linker length or positioning.

Intriguingly, we found that linker length preferences varied between nucleases: the 4-aa linker enhanced FEN1 activity, likely because it requires close proximity to gDNA for flap cleavage, whereas HEX-N2 performed better with the 16-aa linker, possibly due to the need for greater flexibility to excise nucleotides from the flap terminus.

While both EXO1 and FEN1 initially showed promising editing efficiency, only FEN1-containing systems maintained statistically significant improvement over both negative controls and standard PEmax (with 4-FEN demonstrating ~2-fold enhancement). Chen et al. reported FEN1 inhibition reducing editing efficiency, which strengthens our conclusions [8].

In a previous study, we designed and tested 24 different epegRNAs for two different PE generations—PEmax and PE2-NG. We chose PE2-NG because its Cas9 recognizes NG PAM, allowing more freedom in selecting pegRNA variable parts. We only managed to design one spacer sequence for PEmax with very low GC content and some variations in spacer sequences for PE2-NG. We showed that pegRNAs selected for PEmax and co-transfected with the system did not show high stable efficiency, while some of those selected for PE2-NG showed efficiency up to 24% on average in basal airway cells [17]. In this study, we transitioned from epegRNA#1 to epegRNA#2 for evaluating optimal modifications due to inconsistent editing efficiency with the epegRNA#1. This decision was based on prior screening, where epegRNA#2 demonstrated stable and comparatively high editing rates, making it a more reliable candidate for systematic optimization.

We assessed potential undesired effects at both the target editing site and predicted off-target loci. Initial analysis of on-target edits was performed using deep targeted sequencing, which revealed a high background artifact rate likely connected to library preparation [18]. While statistically significant differences between edited samples and negative controls were detected, detailed examination of the mutation spectra showed no meaningful distinctions between edited and non-transfected controls. No mutation patterns suggestive of nuclease-specific activity were observed. Due to inherent technical noise, we could not definitively differentiate true indels from artifacts. However, all observed variants occurred at frequencies no higher than baseline control levels. Notably, only two concurrent SNVs were detected in reads with successful insertions, both at frequencies below 0.3%.

Off-target analysis showed no statistically significant differences in variant rates between edited and control samples across examined loci. Comprehensive evaluation of mutation profiles again revealed no systematic differences attributable to the editing process.

Transient overexpression of FLAG-tagged FEN1 in 293T cells induced DNA damage markers, including γH2AX and RPA32-S4/S8 phosphorylation, along with activation of Chk1 and Chk2 checkpoint kinases [19]. Also, overexpression of both FEN1 and EXO1 is a prognostic marker for a variety of tumors [20,21,22,23]. Although we initially hypothesized that incorporating exonucleases might cause potential risks (prompting our use of optimized linkers), it was critical to empirically assess whether our editing system induced genomic instability. To evaluate this, we performed immunofluorescence staining of γH2AX foci post-editing. We performed the experiment on HEK293T cells as they can be transfected with nearly 100% efficiency, when basal cells only reach about 15% on average and the low efficiency would substantially impair the accurate measurement of γH2AX levels. Our results revealed that cells after 4-FEN lipofection did not differ from control cells in the number of γH2AX foci, suggesting that the system does not increase the likelihood of cancer development.

At the moment, several versions of prime editing have been developed. First, Liu et al. introduced changes in the nCas9 and RT and named the updated system PE2. Later, the team further upgraded the PE2 system; they added NLSs and changed the RT to a codon-optimized version, calling this updated system PEmax. Then they used an additional single guide RNA molecule (sgRNA) to nick the non-edited strand at a location away from the pegRNA target and called the combination of PE2 and sgRNA PE3. PE4 consists of PE2 and MLH1dn, a protein that inhibits the mismatch repair system. PE5 combines PE2, sgRNA, and MLH1dn [8]. PE6 has a truncated version of RT for better delivery, but its efficiency is lower than that of PEmax [24]. PE7 is the most recent modification, in which the small RNA-binding protein LA is fused to the prime editor [25]. In addition, there are several prime editor improvements involving different proteins developed by other scientific groups [26,27,28]. Lastly, some improvements in the pegRNA structure were introduced: Nelson et al. developed engineered pegRNA (epegRNA), which has a 3′-end protection—a structured RNA motif called tevopreQ1 [11]. To minimize potential off-target effects, we directly fused nucleases to the PEmax system without employing additional sgRNAs. Comprehensive genomic stability assays confirmed the safety profile of our constructs. While 4-FEN demonstrated rather low editing efficiency at the *CFTR* locus, its high specificity and favorable safety profile make it a promising candidate for therapeutic development.

Our results vary from a recent study demonstrating enhanced editing through bacteriophage 5′-3′ exonuclease recruitment to the different PE systems, in which researchers managed to increase editing efficiency by adding an aptamer-based recruiter PP7 to the pegRNA (mean increase of 14.4 percentage points averaged across loci compared to PE2, 12.55 percent compared to PE4), while in our study, EXO1 fused to the PEmax system did not show stable efficiency improvement. Authors declined the configuration with exonuclease fused directly to the PE system, as they suggest that both termini of spCas9 are located on the opposite sides of the cleft where the target DNA is bound and nicked, so the efficiency of the exonuclease-PE complex may not show better performance. This may be the reason why our EXO1-based modification did not work properly, but it does not explain FEN1-based modification superiority over standard PEmax [29].

As noted previously, editing AT-rich genomic regions containing pathogenic variants—such as the F508del mutation in *CFTR* that is critically important for gene therapy development—presents particular challenges. Despite the 4-FEN system improvement over PEmax, the achieved F508del correction rate (up to 2.13%) remains below the estimated 10% threshold for clinical efficacy [30]. Notably, our parallel study with PE2-NG systems achieved up to 24% correction through optimized epegRNA design, though delivery limitations persist as the critical bottleneck [17].

Sousa et al.’s recent high-efficiency F508del correction achieved remarkable efficiency (up to 51% in the 16HBEge-F508del cells) through supplemental gRNAs and MLHdn co-delivery; the concomitant increase in editing locus indels (up to 13%) raises safety concerns for therapeutic development [31]. In contrast, our system demonstrated minimal on-target or off-target aberrations, suggesting particular suitability for genomic targets with balanced GC content, but achieved very low F508del correction rates. This observation one more time highlights the importance of finding the correct approach for F508del-caused cystic fibrosis causal treatment.

## 4. Materials and Methods

### 4.1. Plasmid Assembly via Gibson Cloning

The PEmax plasmid backbone (pCMV-PEmax plasmid was a kind gift from David Liu (Addgene plasmid #174820; http://n2t.net/addgene:174820 (accessed on 10 July 2021); RRID:Addgene_174820) was modified by inserting a nuclease and linker sequences using Gibson Assembly (Thermo Fisher Scientific, Waltham, MA, USA). Design was made with the web tool Benchling (https://benchling.com (accessed on 10 July 2025)). Briefly, the PEmax vector and insert fragments with FEN1 or EXO1 were amplified by PCR (Platinum™ SuperFi II PCR Master Mixes, Invitrogen, Carlsbad, CA, USA), purified (Cleanup Mini Kit, Evrogen, Moscow, Russia), and assembled with overlapping ends. The Gibson reaction mix (Gibson Assembly^®^ Master Mix, NEB, Ipswich, MA, USA) contained 5′-exonuclease, DNA polymerase, and DNA ligase, incubated at 50 °C for 15 min. The assembled plasmid was transformed into *E. coli* via heat shock. Positive colonies were screened by PCR (Taq Turbo Buffer, dNTPs, Taq polymerase; Evrogen, Russia) targeting the insert-plasmid junctions (Table A1 and Table A2). Amplicons were verified by agarose gel electrophoresis and Sanger sequencing using the same primers. All of the primers’ sequences used for Gibson cloning are in the Supplementary Materials (Tables S1 and S2).

### 4.2. Derivation of Basal Cells from hiPSCs

Airway basal cells (BCs) were differentiated from induced pluripotent stem cells (hiPSCs) derived from cystic fibrosis (CF) patients homozygous for the *CFTR* F508del variant (patients P1, P5, and P7) [32,33,34]. All participants provided written informed consent (approved by the RCMG Ethical Committee, Protocol #1, 01/28/2016). BCs were cultured in PneumaCult™-Ex Plus Medium (StemCell Technologies, Vancouver, BC, Canada) supplemented with 1 µM A83-01 and 1 µM DMH1 (Tocris Bioscience, Bristol, UK), as previously described [35].

### 4.3. Lipofection of Basal Cells

Transgene delivery was optimized using Lipofectamine LTX (Thermo Fisher). To optimize transfection, different plasmid DNA and Lipofectamine LTX amounts were tested in BCs. Cell viability significantly decreased with higher Lipofectamine LTX concentrations (5 μL), while 2.5 μL maintained viability without compromising efficiency. The highest transfection efficiency was achieved with 0.7 μg plasmid DNA and 2.5 μL Lipofectamine LTX for 24-well plate and 60 × 10^3^ cells/well; Lipofectamine 3000 was used at the manufacturer’s recommended dose for maximal efficiency. For primary screening, two *CFTR* F508del/F508del BC lines (P1L5, P7L2) with 20 × 10^3^ cells/well in 48-well plates were transfected with 350 ng pDNA and 1.25 µL of Lipofectamine LTX. For further experiments, three *CFTR* F508del/F508del BC lines (P1L5, P5L2, and P7L2) with 40 × 10^3^ cells/well in 24-well plates were transfected with 700 ng plasmid DNA and 1.5 µL Lipofectamine 3000 (Thermo Fisher). Transfection efficiency was assessed 72 h post-lipofection by flow cytometry (CytoFLEX, Beckman Coulter, Brea, CA, USA) using GFP expression from a control plasmid (AAT-PB-CG2APtk F508del, 10,067 bp); transfection efficiency values and imaging data (BioTek Lionheart FX Automated Microscope, Aligent, Las Vegas, NV, USA) are provided in Tables S1–S3, Figures S1 and S2, and in the File S1.zip archive). Normalized efficiency = (correction efficiency assessed by CRISPResso2 version 2.2.12) × coefficient, where coefficient = 100%/A, and A is the average transfection efficiency calculated from three technical replicates of GFP-transfected cells by flow cytometry.

### 4.4. PegRNA Design

Two previously validated epegRNAs (epegRNA#1 and epegRNA#2) were used for prime editing. Their sequences are provided in the Table A3 [17].

### 4.5. Editing Efficiency Analysis and Analysis of Unintended Changes in the Editing Site

Genomic DNA was extracted by phenol-chloroform extraction 72 h post-lipofection. The *CFTR* target region was amplified with primers 5F (5′-TGGAGCCTTCAGAGGGTAAAAT-3′) and 8R (5′-TGGCATGCTTTGATGACGCT-3′). Deep targeted sequencing was performed at the RCMG Core Facility using KAPA HiFi HotStart ReadyMix and KAPA Hyper Prep kits with dual-indexed adapters (KAPA UDI Adapter Plate, Roche, Basel, Switzerland). Libraries were sequenced on an Illumina MiSeq (MiSeq v3 600 kit, Illumina, San-Diego, CA, USA). Editing efficiency and undesirable effects were quantified using CRISPResso2 [36]. All mutation data were compiled into a unified table derived from allele frequency tables generated by CRISPResso2. For insertions and deletions, we applied the following filtering steps: First, we excluded single-read variants. Next, we removed reads containing large deletions (≥28 nucleotides) at the termini, as these were also detected in control samples and most likely were artefacts of sequencing. Remaining insertions and deletions were non-recurrent across technical replicates and never exceeded two reads per variant. Consequently, we concluded that the remaining variants represented technical noise rather than true biological events. For mutations in the editing locus, statistical analysis was performed after filtering out sequences with less than 0.05% reads. Raw data are provided in Table S4 and in the File S1.zip archive.

### 4.6. Off-Target Evaluation

Samples after editing with 4-FEN were evaluated with PEmax as positive and non-transfected cells as negative controls. Potential off-target sites, estimated by the Cas-OFFinder tool [37], were amplified from previously extracted genomic DNA using primers listed in Table A4. Amplicons were subjected to deep targeted sequencing (reagents are listed in the Section 4.5) and analyzed using CRISPResso2. All mutation data were compiled into a unified table derived from allele frequency tables generated by CRISPResso2. We applied the same filtering criteria as used for analyzing unintended events at the editing locus. We then compared variants (deletions, insertions, and substitutions) between edited and control samples and excluded variants present in both groups. The remaining reads were non-recurrent across replicates. Raw data are provided in Table S5 and in the File S1.zip archive.

### 4.7. HEK293T Cultivation

HEK293T cells were cultured in DMEM (PanEco, Moscow, Russia) supplemented with 10% fetal bovine serum (Biosera, Cholet, France), GlutaMAX (Thermo Fisher Scientific, USA), 50 U/mL penicillin, and 50 µg/mL streptomycin (PanEco, Russia).

### 4.8. Lipofection of HEK293T Cells

Transgene delivery was optimized using Lipofectamine 3000 (Thermo Fisher). HEK293T cells (600 × 10^3^ cells/well in a 6-well plate) were mixed with 2800 ng plasmid DNA and 10 µL Lipofectamine. Transfection efficiency was assessed 72 h post-lipofection by flow cytometry (CytoFLEX, Beckman Coulter) using GFP expression from a control plasmid (AAT-PB-CG2APtk F508del, 10,067 bp).

### 4.9. Irradiation

Cells were irradiated with 200 kVp X-rays at dose rates of 0.85 Gy/min (5.0 mA, 1.5 mm Al filter) using an RUB RUST-M1 X-ray irradiator (Diagnostika-M LLC, Moscow, Russia). During irradiation, cells were kept at 37 °C using Lab Armour thermo-beads (Life Technologies, Carlsbad, CA, USA). Immediately after exposure, cells were returned to standard growth conditions and maintained for 30 min prior to fixation.

### 4.10. Immunofluorescence Staining

Cells were fixed on coverslips with 4% paraformaldehyde in PBS (pH 7.4) for 20 min at room temperature for γH2AX staining, followed by two PBS washes. Permeabilization was performed using 0.3% Triton-X100 in PBS (pH 7.4) containing 2% bovine serum albumin (BSA) to block nonspecific antibody binding. The cells were then incubated with primary anti-γH2AX antibody (rabbit anti-H2AX, ab81299, Lot: GR3203642-13; Abcam, Cambridge, UK; 1:800 dilution in PBS with 1% BSA) for 1 h at room temperature. After several PBS washes, cells were incubated for 1 h with secondary antibody (goat anti-rabbit IgG (H+L) Alexa Fluor 555, ab150086, Lot: GR3461004-1; Abcam, UK; 1:2000 dilution in PBS with 1% BSA). Finally, coverslips were washed multiple times with PBS and mounted on microscope slides using ProLong Gold Antifade Mountant with DAPI (Life Technologies, USA) for nuclear counterstaining. Cells were viewed and imaged using fluorescence microscopy with an Axio Imager 2 microscope (Carl Zeiss, Oberkochen, Germany). The obtained images were analyzed using FISHView 8.0 software (Applied Spectral Imaging). Images can be found in the File S1.zip archive.

### 4.11. Statistical Analysis

All quantitative data were analyzed using GraphPad Prism 9. Pairwise comparisons between experimental groups were performed using Dunn’s test (a non-parametric post hoc analysis following the Kruskal–Wallis test), which accounts for multiple comparisons by adjusting *p*-values via the Benjamini–Hochberg method. This test was selected due to non-normal data distributions. For visual clarity, figures display mean ± SEM (standard error of the mean), though Dunn’s test utilizes median-based quartile ranges for its calculations.

To quantify the effect size of observed differences, Cliff’s Delta (Δ) was computed using Python 3.12.0, cliffs-delta library. This non-parametric metric ranges from −1 to +1, where Δ > 0.147 indicates a small effect; Δ > 0.33, a medium effect; and Δ > 0.474 a large effect.

Results were considered significant at *p* < 0.05. Also 99th percentile was used in this study for the analysis of undesirable effects in the editing locus.

## 5. Conclusions

While our modified 4-FEN system demonstrated higher editing efficiency compared to PEmax and other variants in targeting the F508del locus, the overall correction rates remained low. This underscores the critical importance of pegRNA optimization for AT-rich genomic regions like *CFTR*. However, the improved performance and lack of oncogenic potential of 4-FEN suggest its potential utility for editing less challenging genomic targets. Future study should therefore pursue two parallel strategies: (1) development of specialized pegRNAs for difficult AT-rich sequences and (2) further optimization of 4-FEN’s architecture, as its enhanced activity may prove particularly valuable for more accessible genomic regions.

## Data Availability

Data available in the Supplementary Materials.

## Supplementary Materials

The supporting information can be downloaded at: https://drive.google.com/drive/folders/1HmOumhs08g-5dpZwWe6STL6Tshn52jj8?usp=drive_link (accessed on 6 July 2025).

## Abbreviations

The following abbreviations are used in this manuscript:

epegRNA	Engineered pegRNA
FEN1	Flap endonuclease
EXO1	Exonuclease 1
RT	Reverse transcriptase
PE	Prime editing

## Appendix A

**Table A1:** Primers for amplification of DNA parts for Gibson cloning.

Name	Direction	Sequence
4-FEN insert	Forward	TTGAATTCCAGAACCACCAGAAGGGGAGGAGTTTTCAATCA
	Reverse	TTTTGAGCCGCCAGATTTTCCCCTTTTAAACTTCCCTGC
4-FEN backbone 1	Forward	GCAGGGAAGTTTAAAAGGGGAAAATCTGGCGGCTCAAAAAGAAC
	Reverse	TTCTGGCCGCCAAGAACCTGTCCGACGCCATCCTGCTGAG
4-FEN backbone 2	Forward	CTCAGCAGGATGGCGTCGGACAGGTTCTTGGCGGCCAGAA
	Reverse	TTGAATTCCAGAACCACCAGAAGGGGAGGAGTTTTCAATCA
FEN-4 insert	Forward	GTCACCAAAGAAGAAGCGGAAAGTCGGAATTCAAGGCCTGGCC
	Reverse	CCAGGCCGATGCTGTACTTCTTGTCAGAACCACCAGATTTTCCCCTTTTAAACTTCCCTGC
FEN-4 backbone 1	Forward	GAAACCTGGGATATAGGGCATC
	Reverse	GACTTTCCGCTTCTTCTTTGG
FEN-4 backbone 2	Forward	GACAAGAAGTACAGCATCGGC
	Reverse	TGCTTCTGACAGATCTGGGC
16-FEN insert	Forward	TCAGGATCAGAAACTCCAGGTACTTCTGAATCTGCTACTCCTGAATCTGGAATTCAAGGCCTGGCCAAA
	Reverse	TTTTGAGCCGCCAGATTTTCCCCTTTTAAACTTCCCTGC
16-FEN backbone 1	Forward	GCAGGGAAGTTTAAAAGGGGAAAATCTGGCGGCTCAAAAAGAAC
	Reverse	TTCTGGCCGCCAAGAACCTGTCCGACGCCATCCTGCTGAG
16-FEN backbone 2	Forward	CTCAGCAGGATGGCGTCGGACAGGTTCTTGGCGGCCAGAA
	Reverse	ACCTGGAGTTTCTGATCCTGAAGGGGAGGAGTTTTCAAT
FEN-16 insert	Forward	GTCACCAAAGAAGAAGCGGAAAGTCGGAATTCAAGGCCTGGCC
	Reverse	CAGATTCAGAAGTACCTGGAGTTTCTGATCCTGATTTTCCCCTTTTAAACTTCCCTGCT
FEN-16 backbone 1	Forward	GAAACCTGGGATATAGGGCATC
	Reverse	GACTTTCCGCTTCTTCTTTGG
FEN-16 backbone 2	Forward	GGTACTTCTGAATCTGCTACTCCTGAATCTGACAAGAAGTACAGCATCGGC
	Reverse	TGCTTCTGACAGATCTGGGC
4-EXO insert	Forward	ACTCCTCCCCTTCTGGTGGTTCTGGGATACAGGGATTGCTACA
	Reverse	GGTTCTTTTTGAGCCGCCAGAGAATTTTTTAAATCCAAA
4-EXO backbone 1	Forward	TTTGGATTTAAAAAATTCTCTGGCGGCTCAAAAAGAACC
	Reverse	TTCTGGCCGCCAAGAACCTGTCCGACGCCATCCTGCTGAG
4-EXO backbone 2	Forward	CTCAGCAGGATGGCGTCGGACAGGTTCTTGGCGGCCAGAA
	Reverse	TGTAGCAATCCCTGTATCCCAGAACCACCAGAAGGGGAGGAGT
EXO-4 insert	Forward	GTCACCAAAGAAGAAGCGGAAAGTCGGGATACAGGGATTGCTACAAT
	Reverse	AGGCCGATGCTGTACTTCTTGTCAGAACCACCAGAGAATTTTTTAAATCC
EXO-4 backbone 1	Forward	GAAACCTGGGATATAGGGCATC
	Reverse	GACTTTCCGCTTCTTCTTTGG
EXO-4 backbone 2	Forward	TGCTTCTGACAGATCTGGGC
	Reverse	TGCTTCTGACAGATCTGGGC
16-EXO insert	Forward 1	ATTGAAAACTCCTCCCCTTCAGGATCAGAAACTCCAGGTACTTCTGAATCTGCTAC
	Forward 2	CAGGTACTTCTGAATCTGCTACTCCTGAATCTGGGATACAGGGATTGCTACAAT
	Reverse	GGTTCTTTTTGAGCCGCCAGAGAATTTTTTAAATCCAAA
16-EXO backbone 1	Forward	TTTGGATTTAAAAAATTCTCTGGCGGCTCAAAAAGAACC
	Reverse	TTCTGGCCGCCAAGAACCTGTCCGACGCCATCCTGCTGAG
16-EXO backbone 2	Forward	CTCAGCAGGATGGCGTCGGACAGGTTCTTGGCGGCCAGAA
	Reverse	ACCTGGAGTTTCTGATCCTGAAGGGGAGGAGTTTTCAAT
EXO-16 insert	Forward	GTCACCAAAGAAGAAGCGGAAAGTCGGGATACAGGGATTGCTACAAT
	Reverse	GGTACTTCTGAATCTGCTACTCCTGAATCTGACAAGAAGTACAGCATCGGC
EXO-16 backbone 1	Forward	GAAACCTGGGATATAGGGCATC
	Reverse	GACTTTCCGCTTCTTCTTTGG
EXO-16 backbone 2	Forward	GACAAGAAGTACAGCATCGGC
	Reverse	TGCTTCTGACAGATCTGGGC
4-HEX insert	Forward	ACTCCTCCCCTTCTGGTGGTTCTGGGATACAGGGATTGCTACA
	Reverse	ACTTGGATTTTCCTTCAATTGTGG
4-HEX backbone 1	Forward	ATTGAAGGAAAATCCAAGTTCTGGCGGCTCAAAAAGAACCGCCGA
	Reverse	TTCTGGCCGCCAAGAACCTGTCCGACGCCATCCTGCTGAG
4-HEX backbone 2	Forward	CTCAGCAGGATGGCGTCGGACAGGTTCTTGGCGGCCAGAA
	Reverse	TGTAGCAATCCCTGTATCCCAGAACCACCAGAAGGGGAGGAGT
HEX-4 insert	Forward	GTCACCAAAGAAGAAGCGGAAAGTCGGGATACAGGGATTGCTACAAT
	Reverse	GCTGTACTTCTTGTCACTACCGCCGGAACTTGGATTTTCCTTCAAT
HEX-4 backbone 1	Forward	GAAACCTGGGATATAGGGCATC
	Reverse	GACTTTCCGCTTCTTCTTTGG
HEX-4 backbone 2	Forward	GACAAGAAGTACAGCATCGGC
	Reverse	TGCTTCTGACAGATCTGGGC
16-HEX insert	Forward 1	ATTGAAAACTCCTCCCCTTCAGGATCAGAAACTCCAGGTACTTCTGAATCTGCTAC
	Forward 2	CAGGTACTTCTGAATCTGCTACTCCTGAATCTGGGATACAGGGATTGCTACAAT
	Reverse	ACTTGGATTTTCCTTCAATTGTGG
16-HEX backbone 1	Forward	ATTGAAGGAAAATCCAAGTTCTGGCGGCTCAAAAAGAACCGCCGA
	Reverse	TTCTGGCCGCCAAGAACCTGTCCGACGCCATCCTGCTGAG
16-HEX backbone 2	Forward	CTCAGCAGGATGGCGTCGGACAGGTTCTTGGCGGCCAGAA
	Reverse	ACCTGGAGTTTCTGATCCTGAAGGGGAGGAGTTTTCAAT
HEX-16 insert	Forward	GTCACCAAAGAAGAAGCGGAAAGTCGGGATACAGGGATTGCTACAAT
	Reverse	AGATTCAGGAGTAGCAGATTCAGAAGTACCTGGAGTTTCTGATCCTGAACTTGGATTTTCCTTCAATTGTGG
HEX-16 backbone 1	Forward	GAAACCTGGGATATAGGGCATC
	Reverse	GACTTTCCGCTTCTTCTTTGG
HEX-16 backbone 2	Forward	GGTACTTCTGAATCTGCTACTCCTGAATCTGACAAGAAGTACAGCATCGGC
	Reverse	TGCTTCTGACAGATCTGGGC

**Table A2:** Primers for colony testing after Gibson assembly. X—4-aa or 16-aa linker.

Name	Direction	Sequence
X-FEN texting	Forward	ACACTCCGCCGAGGCAAG
	Reverse	GCCGTTCTCCATCATGCGAAT
FEN-X testing	Forward	AGGCGTGTACGGTGGGAG
	Reverse	GCCGTTCTCCATCATGCGAAT
X-EXO testing	Forward	ACACTCCGCCGAGGCAAG
	Reverse	ATAGGCTTGATCCCGTGAGA
EXO-X testing	Forward	AGGCGTGTACGGTGGGAG
	Reverse	ATAGGCTTGATCCCGTGAGA
X-HEX testing	Forward	ACACTCCGCCGAGGCAAG
	Reverse	ATAGGCTTGATCCCGTGAGA
HEX-X testing	Forward	AGGCGTGTACGGTGGGAG
	Reverse	ATAGGCTTGATCCCGTGAGA
Backbone testing 1	Forward	GAGTGGAGGGACCCTGAGAT
	Reverse	TGGGATGAACAGCCTGCAAA
Backbone testing 2	Forward	CCGAGGATGCCAAACTGCAGCT
	Reverse	GCCGTTGTCGAAGGTCCG

**Table A3:** epegRNA sequences.

epegRNA#1	GACCATTAAAGAAAATATCATGTTTTAGAGCTAGAAATAGCAAGTTAAAATAAGGCTAGTCCGTTATCAACTTGAAAAAGTGGCACCGAGTCGGTGCACCAAAGATGATATTTTCTTTATACCAACTCGCGGTTCTATCTAGTTACGCGTTAAACCAACTAGAA
epegRNA#2	GACCATTAAAGAAAATATCATGTTTTAGAGCTAGAAATAGCAAGTTAAAATAAGGCTAGTCCGTTATCAACTTGAAAAAGTGGCACCGAGTCGGTGCCACCAAAGATGATATTTTCTTTAACTACCCAGCGCGGTTCTATCTAGTTACGCGTTAAACCAACTAGAA

**Table A4:** Primers for off-target evaluation.

Gene	Forward	Reverse
*IL1RAP*	CTCGCAATGAGGTTTGGTGG	TCATTGACTAGAACACACATCAGAG
*TOX2*	AGTCCTATCCTCAATGCGTGT	TGAGTACTTTTCTTCCTACAGGTCA
*GYPE*	GAAAGAGGCCAAAATCATGTTTATTTAAT	CAACTCTTACTGGCTTAGATGTCA
*WNK2*	CGCTAGAAACAAATGAAACAGAAG	GGGCTGCACACATACTAACTG
*ARID4A*	TTTAAATGGCCTTCCCTAAGACT	TCAACTGTGATAACTATAGAAGTCCCT
*BTLA*	ATATCCCTTGTTAAGTCACTATAGGT	ATGTATAGAGAAAGGGGTTGCTCAAATG
